# When exhaustion meets permissiveness: a response surface analysis of parental burnout–parenting style interactions on childhood social anxiety

**DOI:** 10.3389/fped.2025.1640094

**Published:** 2025-09-10

**Authors:** Peiyi Yang, Lin Yang, Xuerong Liu, Zhengzhi Feng

**Affiliations:** ^1^ Department of Mental Health Education, Chongqing Medical and Pharmaceutical College, Chongqing, China; ^2^ Shanhu Experimental Primary School, Chongqing, China; ^3^ Department of Neurology, Second Affiliated Hospital of Army Medical University (Xinqiao Hospital), Chongqing, China; ^4^ Department of Medical Psychology, Army Medical University, Chongqing, China

**Keywords:** parenting style, parental burnout, child social anxiety, response surface analysis, permissiveness

## Abstract

**Objective:**

This study examined the interactive effects of parental burnout and parenting styles (authoritative, authoritarian, and permissive) on childhood social anxiety using response surface analysis (RSA).

**Methods:**

This cross-sectional study was conducted between November and December 2024 in primary schools in Chongqing, China. Participants (parents and their children) were required to complete online questionnaires, including the Parental Burnout Assessment (PBA), the Parenting Styles and Dimensions Questionnaire (PSDQ), and the Social Anxiety Scale for Children (SASC). RSA and polynomial regression models examined the non-linear interactions between parental burnout, parenting styles, and childhood social anxiety.

**Results:**

A total of 724 datasets were included in the study. The findings indicated that significant congruence effects emerged for permissive parenting and parental burnout, and an inverted U-shaped curvature appeared along the line of incongruence with childhood social anxiety (curvature, a4 = −0.70, *p* = 0.009; slope a3 = −0.57, *p* = 0.272). Moreover, the curvature (a2 = −0.28, *p* = 0.089) and slope (a1 = 0.81, *p* = 0.068) were measured along the line of congruence, indicating that the line of congruence section curve is linearly rising.

**Conclusion:**

This study identifies a significant interactive effect between parental burnout and permissive parenting style on childhood social anxiety, highlighting the need for two-pronged interventions. Future research should investigate the longitudinal causal pathways between parental burnout–parenting style congruence and child social anxiety across diverse cultural contexts.

## 1 Introduction

From childhood through adolescence, individuals exhibit heightened sensitivity to social feedback, including peer evaluations and interpersonal performance, reflecting the natural progression of cognitive and social competencies. Although transient social apprehension during this developmental stage is normative, persistent escalation of anxiety beyond adaptive coping capacities may precipitate social anxiety in susceptible children (1). This prevalent pediatric psychological disorder is characterized by intense fear during social interactions or performance contexts, accompanied by excessive concerns regarding negative evaluation. It manifests through maladaptive avoidance behaviors (e.g., public speaking difficulties, peer engagement avoidance, or inhibited friendship initiation) (2, 3) and physiological symptoms such as crying, blushing, panic reactions, or emotional outbursts (4). Epidemiological data from Chavira et al. (5) indicated that approximately 19% of children exceed clinical thresholds for social anxiety. Critically, this condition extends beyond transient distress to inflict long-term mental health impairments, heightening risks for social withdrawal, academic dysfunction, comorbid depression, diminished self-esteem, and suicidal ideation in their future development (6, 7). Moreover, affected children reported more frequent and severe somatic complaints such as gastrointestinal disturbances, cardiovascular symptoms, and fatigue compared with peers (8, 9). To develop effective interventions for children with social anxiety and their parents, clinicians and researchers need to identify determinants of pediatric social anxiety, particularly modifiable environmental factors.

Bronfenbrenner's ecological systems theory (10) posits that early psychological development is shaped by multi-layered environmental systems, with the family environment constituting a pivotal influence. This framework emphasizes familial contexts as both primary arenas for early social experiences and critical buffers against external stressors. Consequently, familial factors represent key intervention targets for pediatric social anxiety. Research suggested parenting styles modulate children's social cognitive schema and social anxiety susceptibility through three core mechanisms: emotional responsiveness, rule-setting efficacy, and behavioral monitoring (11, 12). Baumrind's classification (13) delineates three parenting archetypes: authoritative (emotional support with autonomy-promoting guidance), authoritarian (behavioral compliance with limited emotional engagement), and permissive (high acceptance without requisite behavioral guidance). Recent studies demonstrated differential associations between these styles and pediatric social anxiety. Pinquart (14) revealed heightened permissive tendencies among parents of anxious children, suggesting that ambiguous rule structures may exacerbate symptoms. Lei et al. (15) elucidated stronger associations between negative parenting practices and social anxiety in Asian vs. Western contexts. Conversely, authoritarian, authoritative, and overprotective parenting styles are associated with reduced social anxiety (16, 17).

In addition, parental burnout, defined as “emotional exhaustion, reduced parenting motivation, and emotional distance resulting from chronic parenting stress” (18), has garnered empirical attention. In the Chinese cultural context, 35% of parents reported chronic burnout stemming from occupation, parenting, and child education demands (19, 20). Previous research claimed that children of burned-out parents exhibit heightened susceptibility to social anxiety (21, 22). Exhausted parents often demonstrate diminished capacity to provide appropriate attention and feedback, while experiencing increased familial conflicts that impair children's interpersonal skill development (23, 24). Chronic exposure to such low-warmth home environments may foster persistent insecurity, disrupting social skill acquisition through heightened attention bias toward threatening social cues. This aligns with findings that anxious children exhibited cognitive biases when processing social information, disproportionately attending to negative cues (e.g., critical facial expressions) and overinterpreting threats in ambiguous social situations (25).

Although family-centered interventions for socially anxious children are widely implemented (26–28), their efficacy remains inconsistent. Longitudinal data from Ginsburg et al. (29) indicated that 41% of cases experienced symptom recurrence within 3 years post-intervention, highlighting limitations in current conceptualizations of the parental burnout–parenting style–child social anxiety pathway.

Bronfenbrenner's ecological systems theory (10) suggests synergistic effects between parental factors, implying that burnout and parenting styles may jointly elevate social anxiety risk. However, existing research (14, 15, 19, 20) predominantly examined isolated variables, either parental burnout or permissive parenting, and overlooked their interaction effect on childhood social anxiety. Crucially, no studies have investigated whether congruence between high parental burnout and permissive parenting predicts anxiety severity. Accordingly, we propose that when parental burnout and permissive parenting style co-occur, it critically undermines parental intervention capacity due to “no rules and no support” environments. Consequently, we hypothesize that high parental burnout, coupled with a permissive parenting style, significantly predicts severe pediatric social anxiety.

Our study addressed the gap by utilizing response surface analysis (RSA) to assess how interactions between parental burnout and parenting styles predicted childhood social anxiety, leveraging RSA's capacity to quantify non-linear effects beyond traditional single-factor approaches. This advanced methodology capitalizes on RSA's capacity to quantify non-linear effects beyond traditional single-factor analyses through polynomial regression modeling with quadratic (*X*^2^, *Y*^2^) and interaction (*XY*) terms, thereby enabling identification of complex variable relationships (30). Specifically, we utilized RSA to assess how both congruence (similar levels of parental burnout and parenting styles) and incongruence (divergent levels between these factors) influenced childhood social anxiety. By transcending univariate limitations of conventional regression paradigms, this method elucidated synergistic interactions between parental psychological resources (parental burnout) and caregiver behaviors (parenting styles).

## 2 Materials and methods

### 2.1 Participants and procedure

This cross-sectional study was conducted between November and December 2024 across three public elementary schools in Chongqing, China. Participants were recruited through classroom teachers, and one parent, either the mother or father, was invited to complete an online questionnaire assessing parental burnout and parenting styles. Meanwhile, their 8–12-year-old children independently completed an online survey measuring social anxiety. To minimize comprehension bias and attentional limitations inherent in this age group, this study adopted the Social Anxiety Scale for Children (SASC) with only 10 items. A valid dataset was defined as a complete response and matched data from one parent and their child. To facilitate matching, the child and parent were required to enter the “mother’s name and last 4 digits of phone number” to match the dataset. The study protocol was approved by the institutional ethics committee, and online informed consent was obtained from all participants.

### 2.2 Measures

#### 2.2.1 SASC

The SASC, developed by La Greca and Lopez (31), is a psychometric instrument designed to assess child anxiety experiences in social contexts. This 10-item scale comprises two sub-scales: “fear of negative evaluation” (e.g., I worry that others will laugh at me) and “social avoidance and distress” (e.g., I feel nervous when talking to unfamiliar peers). Each item is rated on a 3-point Likert scale ranging from 0 (never) to 2 (always), with higher values indicating more social anxiety symptoms. In this study, the SASC demonstrated reliability and validity in the Chinese pediatric populations (32), with an overall Cronbach's *α* coefficient of 0.88.

#### 2.2.2 Parental Burnout Assessment

The Parental Burnout Assessment (PBA) is a validated psychometric instrument designed to evaluate burnout symptoms arising from chronic parenting stress (23). This 21-item scale comprises four sub-scales: “exhaustion in one's parental role” (e.g., I'm exhausted by the efforts I have to make to be a good parent), “contrast with previous parental self” (e.g., I no longer know how to be a good parent), “feelings of being fed up with one's parental role” (e.g., I can no longer tolerate my role as a parent), and “emotional distancing from one's children” (e.g., I only do the bare minimum required for my child). Each item is rated on a 7-point Likert scale ranging from 1 (never) to 7 (daily), with higher total scores indicating greater parental burnout severity. In this study, the PBA demonstrated reliability and validity in Chinese parental populations (33), with an overall Cronbach's *α* coefficient of 0.94.

#### 2.2.3 Parenting Styles and Dimensions Questionnaire

The Parenting Styles and Dimensions Questionnaire (PSDQ), designed by Robinson et al. (34), is designed to assess different types of parenting behaviors. The 32-item questionnaire comprises three sub-scales: “authoritative parenting” (e.g., I patiently listen to my child's perspectives and collaboratively establish rules with them), “authoritarian parenting” (e.g., I expect my child to comply with my demands unconditionally), and “permissive parenting” (e.g., I rarely intervene in my child's behavior, even when mistakes may occur). Each item is rated on a 5-point Likert scale ranging from 1 (never) to 5 (always). Sub-scales are computed independently, with higher scores indicating stronger stylistic tendencies. In this study, the PSDQ demonstrated an overall Cronbach's *α* coefficient of 0.83, and sub-scales’ Cronbach's α coefficients of 0.95, 0.89, and 0.69, respectively.

### 2.3 Data analysis

Data analysis was conducted using SPSS 29.0, with RSA implemented via the SPSS RSA plugin (35) following Edwards and Cable's methodological framework (36). Firstly, predictor variables for parental burnout (*X*) and parenting styles (authoritative, authoritarian, or permissive) scores (*Y*) were centralized. Then, a polynomial regression model was constructed using the centralized predictor variables (*X*, *Y*), their squared terms (*X*^2^, *Y*^2^), and product terms (*XY*) as independent variables, with childhood social anxiety (*Z*) as the dependent variable. This tested the curve relationship and interaction association. Subsequently, the appropriateness of RSA was confirmed by a significant *R*^*2*^ increment and at least one statistically significant quadratic term.

The results were interpreted through response surface features such as response surface stationary points, curvature of cross sections of line of congruence (LOC; *X* *=* *Y*, indicating alignment between parental burnout and parenting style) and line of incongruence (LOIC; *X* *=* −*Y*, indicating the divergent levels between parental burnout and parenting style), and the relationship between the first principal axis (FPA) and a1–a5. A three-dimensional response surface was generated from the polynomial regression output, with parameters for RSA a1–a5 were calculated based on the regression coefficients b0–b5 as follows: a1 = b1 + b2 indicates linear slope along LOC, a2 = b3 + b4 + b5 indicates curvature along LOC, a3 = b1–b2 indicates linear slope along LOIC, a4 = b3–b4 + b5 indicates a curvature along LOIC, a5 = b3–b5 indicates an alignment between FPA and LOC.

Humberg et al. (37) specified four sequential conditions to validate congruence effects. FPA must align with the LOC, the FPA intercept (P10) must not differ significantly from 0 (Condition 1), and the FPA slope (P11) must not differ significantly from 1 (Condition 2). The LOIC (*X* *=* *−Y*) curvature (a4) requires significant negative (Condition 3), and the LOIC slope at the origin (a3) must not differ significantly from 0 (Condition 4). Polynomial regression coefficients quantified variable relationships of b1 (parental burnout *X*), b2 (parenting type *Y*), b3 (*X*^2^), b5 (*Y*^*2*^), and b4 (*X* and *Y*).

Common method variation arising from scale type or answer formats may induce common method bias (CMB), potentially causing bias between data results and actual results (38). To assess CMB in our questionnaire-based study, all items from the three scales underwent exploratory factor analysis (EFA) using Harman's single-factor test with non-rotated principal component analysis. The results of this analysis are reported in the “Results” section.

## 3 Results

### 3.1 Demographic characteristics

A total of 1,247 parent questionnaires and 1,049 child questionnaires were collected via the online platform. After excluding 225 unmatched responses and 298 incomplete responses, 724 valid parent–child paired datasets remained for analysis. The demographic characteristics of the participants are presented in Table 1. Among the parent participants, 24.17% had an educational attainment at or below the junior high school level; 21.69% had completed high school or technical secondary school education; 23.2% had junior college education; and 25.69% had an undergraduate education. Regarding marital status, 631 households (87.16%) had married parents, while 57 households (7.87%) had divorced parents. Among the child participants, 49.31% were boys and 50.69% were girls. In addition, 42.68% of the children were the only child in their family. More details are listed in Table 1.

**Table 1:** Demographic characteristics of parent–child sample in Chongqing, China (*n* = 724).

Variable	Characteristic	Sample size	Ratio (%)
Parent age	Under 28 years old	6	0.83
	28–35 years old	171	23.62
	35–45 years old	476	65.75
	45 years old and above	71	9.80
Parent educational background	Junior high school and below	175	24.17
	High school/vocational school	157	21.69
	College degree	168	23.2
	Bachelor's degree	186	25.69
	Postgraduate and above	38	5.25
Child gender	Male	357	49.31
Only child in the family	Yes	309	42.68
Parent marital status	Unmarried	4	0.55
	Married	631	87.16
	Divorced	57	7.87
	Lost a spouse	4	0.55
	Remarried	28	3.87
Living environment	City	624	86.19
	Town	64	8.84
	Rural	36	4.97
Monthly household income	<2,000 Yuan	38	5.25
	2,000–5,000 Yuan	168	23.2
	5,000–10,000 Yuan	264	36.46
	10,000–100,000 Yuan	237	32.74
	Over 100,000 Yuan	17	2.35

### 3.2 Common method variance

Generally, if EFA yields two or more factors with eigenvalues >1 and the variance explained by the first factor is <50%, this indicates that there is no severe CMB (39, 40). Our results identified 12 factors with eigenvalues >1, and the variance explained by the first 22.42% is less than the critical threshold of 40%. These findings indicated no significant CMB in this study.

### 3.3 Descriptive statistics and correlation analysis among variables

Table 2 presents the results of the mean, standard deviation, and correlation between variables. Childhood social anxiety demonstrated significant positive correlations with parental burnout (*r* = 0.200), authoritarian parenting style (*r* = 0.202), and permissive parenting style (*r* = 0.206) and indicated a significant negative correlation with authoritative parenting style (*r* = −0.165).

**Table 2:** Descriptive statistics and correlations between parental burnout, parenting styles, and childhood social anxiety.

Variable	1	2	3	4	5
Parental Burnout	1				
Authoritative	−0.197***	1			
Authoritarian	0.341***	−0.336***	1		
Permissive	0.334***	−0.243***	0.546***	1	
Social anxiety	0.200***	−0.165***	0.202***	0.206***	1
M ± SD	34.48 ± 15.99	59.92 ± 10.78	24.20 ± 6.66	10.95 ± 3.11	15.59 ± 4.04

(1) Parental burnout; (2) authoritative parenting style; (3) authoritarian parenting style; (4) permissive parenting style; (5) childhood social anxiety.

*** *p* < 0.001.

### 3.4 The association between parental burnout and authoritative parenting style on child social anxiety

**Figure 1:**
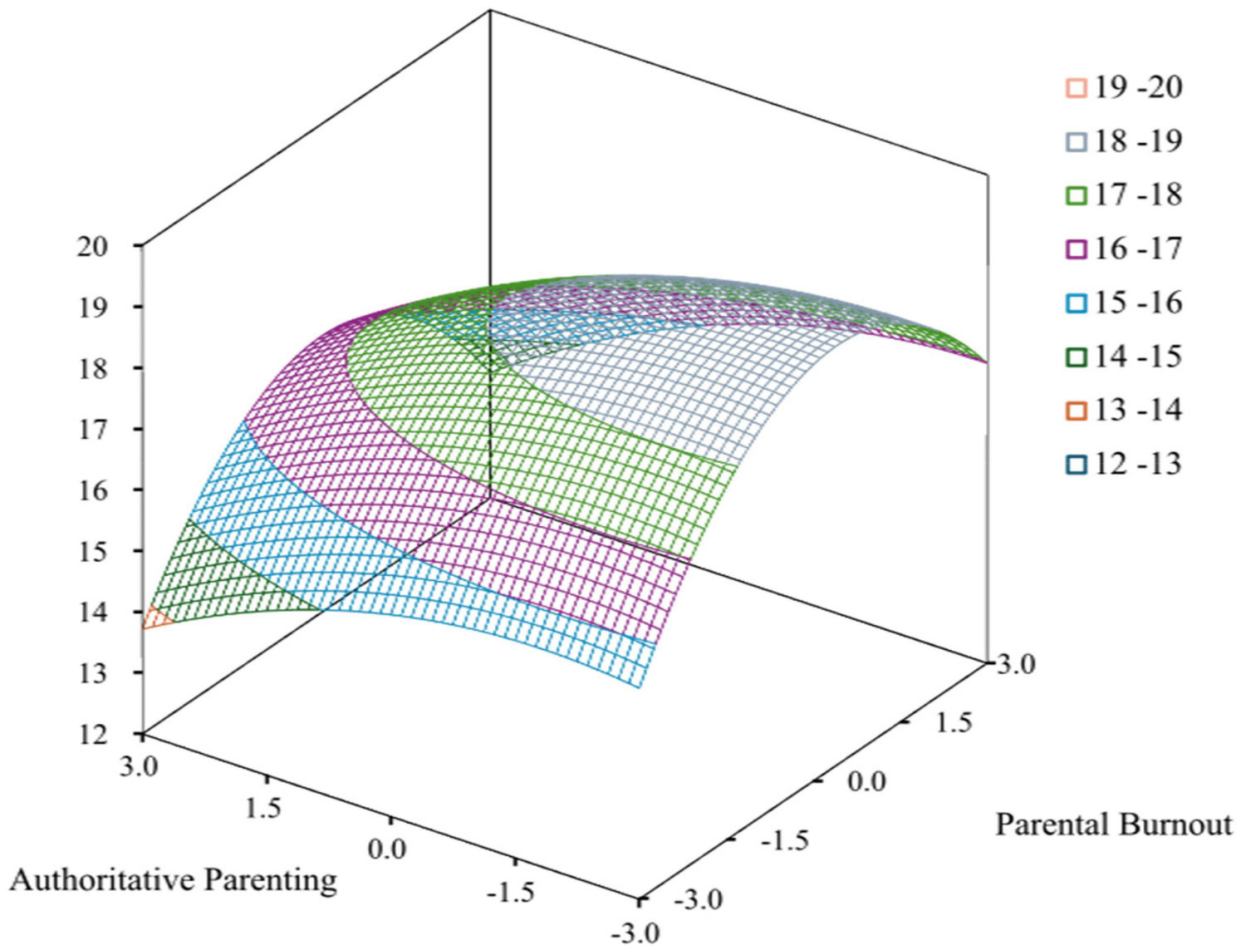
Response surface analysis of parental burnout–authoritative parenting interaction on childhood social anxiety.

Followed the approach of Fleenor et al. (41), level variance analysis (±0.5 SD threshold) revealed the following case distribution: *X* > *Y* (29.83%), *X* = *Y* (26.66%), and *X* < *Y* (43.51%). This relatively uniform distribution supported RSA implementation. The regression results (Table 3, Figure 1) presented that the *R*^2^ of the model was 0.07 (*F* = 10.74, *p* < 0.001), indicating a statistically significant model that captured meaningful variance; hierarchical regression was conducted on three second-order terms (*X*^2^, *Y*^2^, *XY*), and the increment of *R*^2^ was 0.013 (*p* < 0.05). Δ*R*^2^ was significant, and there were statistically significant quadratic terms (*p* < 0.01), validated by subsequent analysis. The RSA results presented a5 = −0.21 (*p* = 0.108), indicating that the first main axis matched with LOC, with the curvature (a4 = −0.34, *p* = 0.058) and slope (a3 = 0.53, *p* = 0.385. As LOIC did not reach statistical significance, parental burnout and authoritative parenting style matches did not significantly predict childhood social anxiety.

**Table 3:** Response surface analysis parameters for parental burnout–authoritative parenting interaction predicting child social anxiety.

Variable	Model 1		Model 2	
	B	*t*	B	*t*
(Constant)	18.20	41.85***	18.39	29.71***
Parental burnout (b1)	0.82	4.73***	0.15	0.48
Authoritative type (b2)	−0.49	−3.54****	−0.38	−0.87
Parental Burnout × parental Burnout (b3)			−0.29	−2.80**
Parental Burnout × authoritative type (b4)			−0.031	−0.18
Authoritative type × authoritative type (b5)			−0.08	−1.06
LOC				
Slope: a1(b1 + b2)			−0.23	−0.52
Curvature: a2(b3 + b4 + b5)			−0.40	−1.64
LOIC				
Slope: a3(b1–b2)			0.53	0.87
Curvature: a4(b3–b4 + b5)			−0.34	−1.90
Principal axis matching				
a5(b3–b5)			−0.21	−1.61
* R* ^2^	0.06		0.07	
* F*	21.59***		10.74***	
Δ*R*^2^			0.013*	

* *p* < 0.05.

*** *p* < 0.001.

### 3.5 The association between parental burnout and authoritarian parenting style on child social anxiety

**Figure 2:**
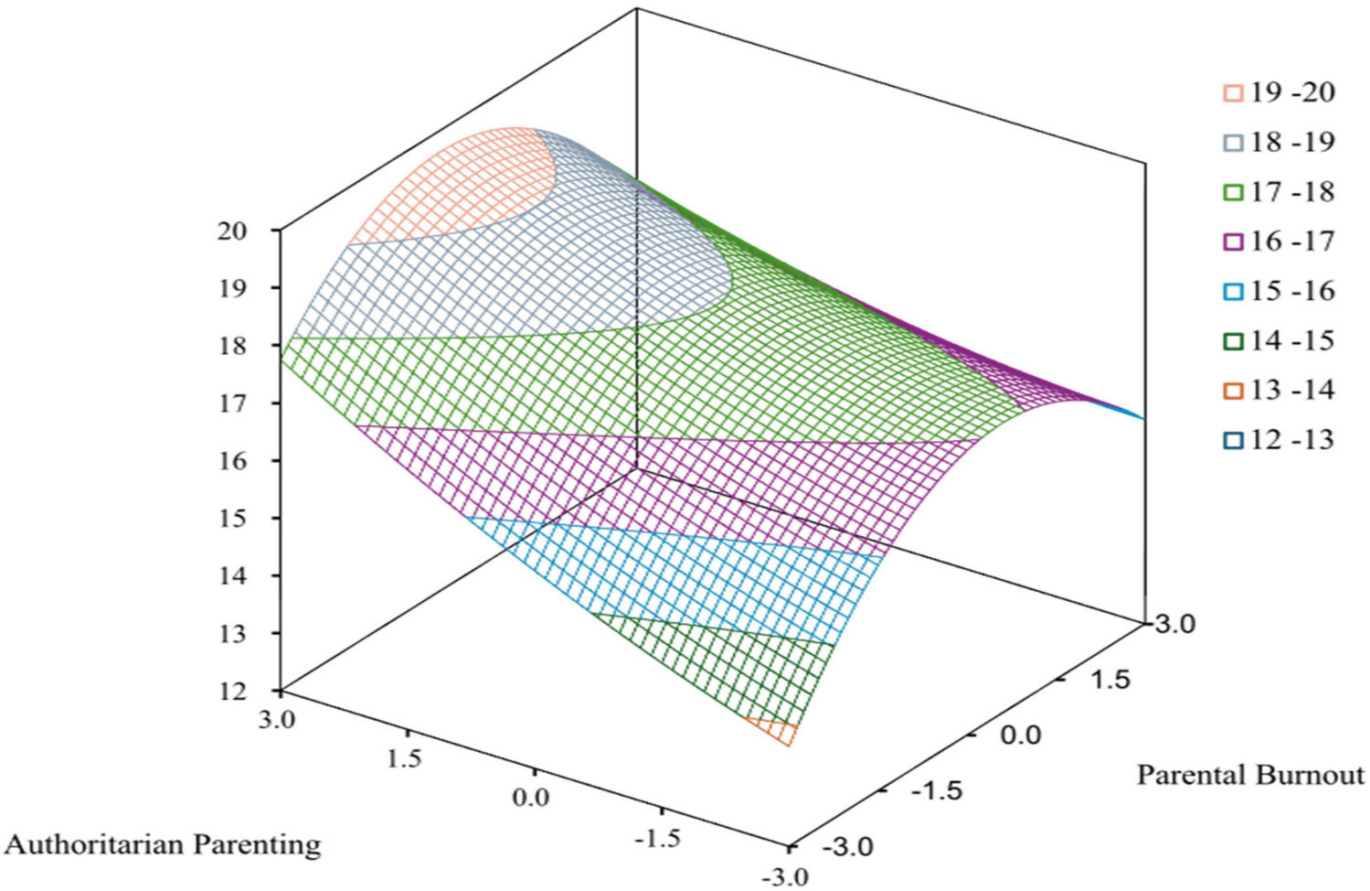
Response surface analysis of parental burnout–authoritarian parenting interaction on childhood social anxiety.

A 0.5 standard deviation threshold was applied for horizontal difference analysis, revealing a balanced case distribution of *X* *>* *Y* (27.35%), *X* *=* *Y* (43.09%), and *X* *<* *Y* (29.56%), supporting the suitability of RSA. As presented in Table 4 and Figure 2, hierarchical regression was conducted on three second-order terms (*X*^2^, *Y*^2^, *XY*), and the increment of *R*^2^ was 0.01 (*p* = 0.062). Δ*R*^2^ was not significant, but there were statistically significant quadratic terms (*p* < 0.01). The RSA results presented a5 = −0.29 (*p* = 0.063), demonstrating that the first principal axis matches the LOC, with the curvature (a4 = −0.15, *p* = 0.59) and slope (a4 = −0.37, *p* = 0.485) along the LOIC were not significant. This pattern violates the congruence assumptions for RSA, and thus, parental burnout–authoritarian parenting style congruence does not significantly predict childhood social anxiety.

**Table 4:** Response surface analysis parameters for parental burnout–authoritarian parenting interaction predicting child social anxiety.

Variables	Model 1		Model 2	
	B	*t*	B	*t*
(Constant)	17.953	42.95***	17.97	37.09***
Parental burnout (b1)	0.702	3.87***	0.077	0.265
Authoritarian type (b2)	0.628	3.94***	0.443	0.981
Parental burnout × parental burnout (b3)			−0.256	−2.42*
Parental burnout × authoritarian type (b4)			−0.075	−0.41
Authoritarian type × authoritarian type (b5)			0.034	0.32
LOC				
Slope: a1(b1 + b2)			0.52	0.94
Curvature: a2(b3 + b4 + b5)			−0.30	−1.60
LOIC				
Slope: a3(b1−b2)			−0.37	−0.70
Curvature: a4(b3−b4 + b5)			−0.15	−0.55
Principal axis matching				
a5(b3−b5)			−0.29	−1.86
* R* ^2^	0.06		0.07	
*F*	23.18***		10.80***	
Δ*R*^2^			0.01	

* *p* < 0.05.

*** *p* < 0.001.

### 3.6 RSA of parental burnout–permissive parenting style on child social anxiety

**Figure 3:**
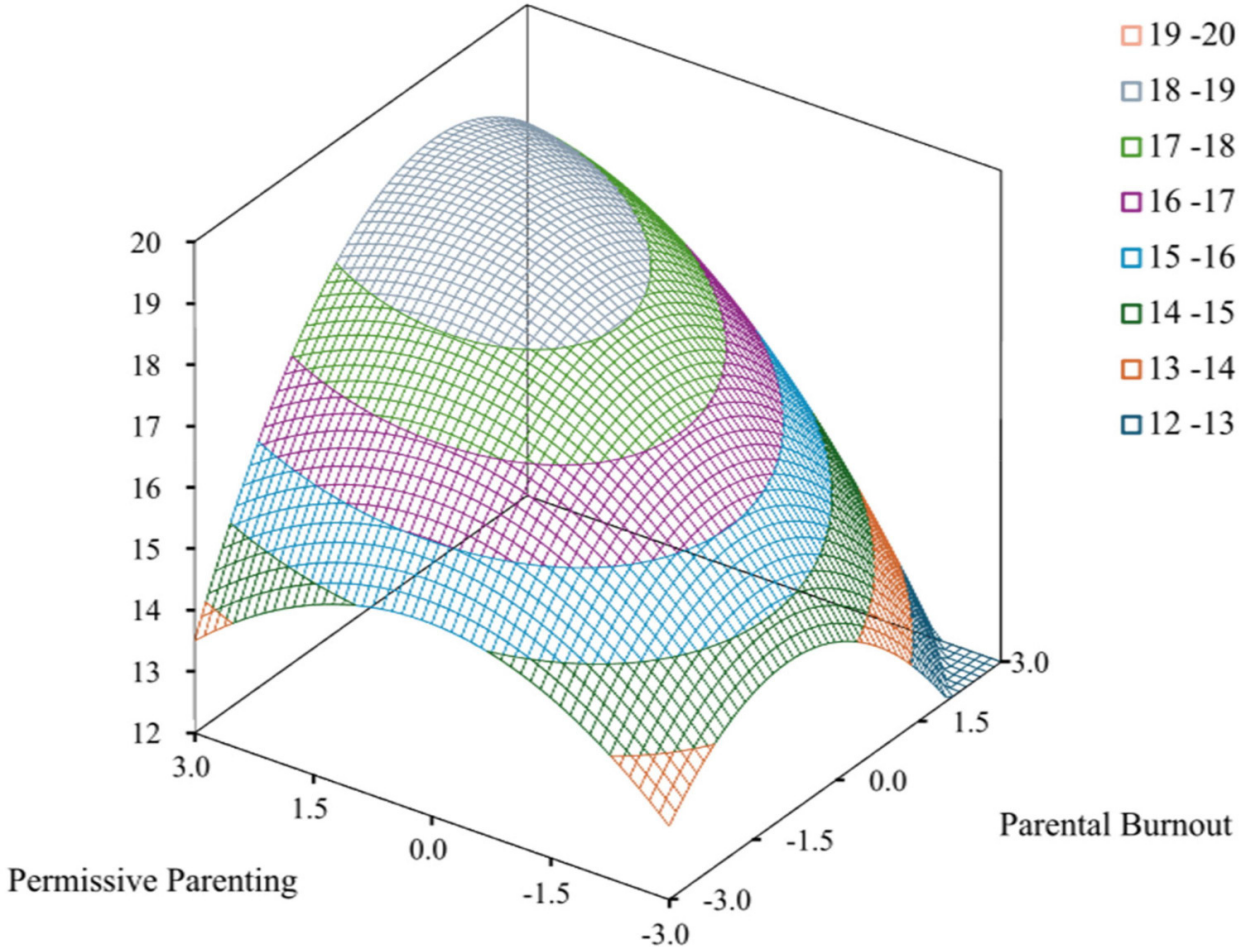
Response surface analysis of parental burnout–permissive parenting interaction on childhood social anxiety.

A threshold of 0.5 standard deviations was adopted for horizontal difference analysis, yielding the following case distribution: *X* *>* *Y* (27.90%), *X* *=* *Y* (38.95%), and *X* *<* *Y* (33.15%), confirming balanced representation for RSA. The results presented in Table 5 demonstrate first principal axis matches with LOC (a5 = −0.09, *p* = 0.526), significant LOIC curvature (a4 = −0.70, *p* = 0.009), and non-significant LOIC slope (a3 = −0.57, *p* = 0.272). The significant negative LOIC curvature indicated an inverted U-shaped cross section with its vertex at the origin (0,0). Along the LOC, neither curvature (a2 = −0.28, *p* = 0.089) nor slope (a1 = 0.81, *p* = 0.068) reached statistical significance, indicating that the LOC section curve was linearly rising. Collectively, these results satisfied strict congruence assumptions, with the response surface ridges line exhibiting an upward trajectory (Figure 3). Specifically, higher congruence between elevated parental burnout levels and permissive parenting styles predicted increased levels of social anxiety in children.

**Table 5:** Response surface analysis parameters for parental burnout–permissive parenting interaction predicting child social anxiety.

Variable	Model 1		Model 2	
	B	*t*	B	*t*
(Constant)	17.70	42.71***	18.11	41.44***
Parental burnout (b1)	0.70	3.87***	0.12	0.43
Permissive type (b2)	0.58	4.10***	0.69	1.80
Parental burnout × parental burnout (b3)			−0.29	−2.71**
Parental burnout × permissive type (b4)			0.21	1.29
Permissive type × permissive type (b5)			−0.20	−2.03*
LOC				
Slope: a1(b1 + b2)			0.81	1.83
Curvature: a2(b3 + b4 + b5)			−0.28	−1.70
LOIC				
Slope: a3(b1−b2)			−0.57	−1.10
Curvature: a4(b3−b4 + b5)			−0.70	−2.63**
Principal axis matching				
a5(b3−b5)			−0.09	−0.63
*R*^2^	0.062		0.077	
*F*	23.83***		11.91***	
Δ*R*^2^			0.015*	

* *p* < 0.05.

** *p* < 0.01.

*** *p* < 0.001.

## 4 Discussion

This study investigated how congruence between parental burnout and parenting styles (authoritative, authoritarian, and permissive) related to childhood social anxiety. RSA revealed that concurrent high levels of parental burnout and permissive parenting predicted significantly elevated childhood social anxiety. Conversely, congruence of authoritative or authoritarian parenting styles with parental burnout demonstrated no statistically significant association with childhood social anxiety.

According to RSA, the high level of parental burnout and permissive parenting congruence manifested a significant inverted U-shaped curvature along the LOIC. Such a pattern indicated that combined elevations in parental burnout and permissive parenting style predicted increased childhood social anxiety.

These findings support our hypothesis and align with existing literature demonstrating that permissive parenting style negatively correlates with children's socioemotional competence (42, 43), while parental stress and dysfunctional parent–child interaction mediate childhood social anxiety development (44, 45). Bandura's social learning theory (46) provides a theoretical framework for these results, positing that children acquire behavioral patterns through observation and imitation of caregivers' emotional and behavioral expressions. This observational learning process is particularly salient during childhood, facilitating the acquisition of social skills and emotional regulation strategies while shaping children's understanding of social dynamics (47). Notably, when parents experience a high level of parental burnout, they exhibit disengagement through emotionally detached or minimally communicative parenting. Under conditions of high parental burnout, caregivers frequently exhibit disengagement through emotionally detached or minimally communicative parenting. Children may subsequently internalize these withdrawal behaviors (e.g., reduced parental responsiveness) and negative affective states (e.g., irritability and anxiety), adopting similar avoidance strategies during peer conflicts. For instance, ambiguous social cues such as peer laughter may be misinterpreted as an unknown threatening stimulus by a child, potentially triggering emotional breakdown such as running away or yelling (48). Thereby, these maladaptive social learning processes amplify children's vulnerability to social anxiety.

Moreover, Bandura's social learning theory emphasizes the critical role of feedback mechanisms in observational learning processes (46). Children in high burnout and permissive households may develop heightened social anxiety due to insufficient parental feedback such as diminished rule-setting and guidance behaviors from parents (49). For instance, when children encounter peer conflicts, permissive and burned parents often fail to provide adequate feedback regarding social norms and appropriate responses. This deficiency may lead children to misinterpret routine social interactions as unpredictable threats, triggering avoidant behaviors (e.g., declining social invitations) and somatic anxiety symptoms (e.g., blushing or panic) (31). Consequently, children raised in such environments with ambiguous behavioral boundaries experience impaired threat perception and social cognition. These distortions prevent accurate interpretation of social cues, thereby exacerbating anxiety symptoms (50, 51). The resulting perceptual biases establish a self-reinforcing cycle wherein maladaptive interpretations potentiate social anxiety pathology.

Furthermore, the congruence between authoritative parenting and parental burnout did not significantly predict childhood social anxiety. This finding is similar to the results reported by some researchers, which indicated that authoritative parenting style reduces children's vulnerability to social anxiety (52, 53). Within China's cultural context, this may be attributed to the resilience of authoritative parenting practices, which emphasize collective values and parental authority. In Chinese culture, authoritative parenting characterized by well-defined rules and rational communication typically persists in a positive parenting behavior even during parental burnout (54). Such parents sustain appropriate behavioral boundaries during high burnout periods (e.g., their children cannot use foul language or hit someone; otherwise, they will be grounded. If others attack their children, then their children should report it to them). This clear social guidance aids children in comprehending interpersonal relationships and establishing clear interaction expectations, thereby buffering uncertainty-induced social anxiety (55). Also, unlike authoritarian approaches, authoritative parents avoid excessive control of their children while offering more opportunities for them to acquire social skills (e.g, peer conflict resolution and self-expression). It may reduce children's excessive concerns about others' evaluations, consequently mitigating social anxiety development. Collectively, although parental burnout may reduce authoritative parents' disciplinary engagement, their pre-established behavioral frameworks continue to provide essential social scaffolding and psychological security.

Moreover, the congruence between authoritarian parenting and parental burnout did not significantly predict childhood social anxiety. This finding contrasts with Western and European research indicating that authoritarian and over-controlling parenting styles as significant predictors of childhood social anxiety (56, 57). This discrepancy may reflect culturally distinct mechanisms underlying Chinese children's response to authoritarian parenting. Within collectivist societies emphasizing group harmony, interdependence, and obedience, parental psychological and behavioral control exhibits different functional outcomes than in individualistic cultures. In the Chinese collectivist context, authoritarian practices are more prevalent and appear to be less detrimental to children compared with Western and European individualistic cultures. Gao et al.'s longitudinal study (58) corroborates this, indicating minimal impact of parental psychological control on Chinese early adolescents' social anxiety. Chinese parents regulate their children's negative behavior by employing guilt-induction strategies grounded in Confucian values (parental self-sacrifice narratives such as I work hard for your future) while emphasizing social consequences of misbehavior. These disciplinary approaches frame control as expressions of concern rather than hostility, potentially mitigating threats and anxiety perceived by children. Critically, parental burnout may attenuate authoritarianism's intensity. Exhausted parents typically reduce coercive control efforts (e.g., punitive demands and physical punishments), inadvertently creating behavioral regulation without excessive domination. This burnout-induced moderation likely counterbalances authoritarianism's anxiety-provoking effects, explaining the non-significant association observed in our study.

In addition, parental mental health further compromises family environmental stability, potentially exacerbating childhood social anxiety through indirect pathways. Existing research confirmed significant associations between parental burnout and anxiety (24, 59). This psychopathological burden impairs caregivers' capacity to maintain emotional regulation, thereby destabilizing the family system. Specifically, burnout manifests through unpredictable parental affect (e.g., sudden anger or emotional withdrawal) (60) and diminishes co-parenting efficacy amid persistent familial conflict (61). Such an unstable and conflict family environment erodes children's emotional security, which is defined as their perceived safety within the familial relationships and observation of parental conflict resolution strategies (62). Parents who experienced burnout while practicing permissive parenting may intensify children's emotional insecurity through burnout-induced household chaos, coupled with permissive parenting's failure to provide children with the necessary guidance for fostering emotional security. When children lack foundational emotional security, they frequently misinterpret external social cues as threats, triggering defensive avoidance behaviors characteristic of social anxiety (63). Therefore, social anxiety symptoms may function as self-protective responses in children who live with burnout and permissive parents.

In general, our findings demonstrated that parents who adopt permissive parenting styles and simultaneously experience parental burnout predicted pediatric social anxiety, revealing the synergistic association between parenting styles and parental burnout in driving childhood social anxiety.

### 4.1 Implications

The study yields two critical implications for family-based interventions targeting childhood social anxiety. First, psychological interventions for parents should implement integrated assessments evaluating concurrent parenting styles and parental burnout levels, with tailored strategies addressing identified risk profiles. For parents exhibiting burnout–permissive parenting style, intervention should prioritize fatigue management through mindfulness and cognitive–behavior therapy, enhance emotional regulation capacities, and develop boundary-setting competencies to mitigate permissive tendencies. Second, a structured parenting training program should establish developmentally appropriate interaction frameworks, including in family interventions, to help permissive parents establish clear daily interaction rules for their children and reduce potential social uncertainties in their interpersonal communication.

### 4.2 Limitations

First, the cross-sectional design of this study restricts causal inferences regarding bidirectional parent–child influences, necessitating future longitudinal investigations using cross-lagged panel models to elucidate temporal dynamics. Second, the regional sampling was restricted to Chongqing, China, highlighting the need for multi-site replications with expanded demographic representation. Third, variable-centered analyses may obscure holistic parenting profiles, suggesting person-centered approaches (e.g., latent profile analysis) as valuable future directions. Lastly, cultural factors (e.g., collectivist values emphasizing obedience) may attenuate the influences of authoritarian parenting. Future researchers should replicate the study in individualistic contexts to examine its cultural generalization.

## 5 Conclusion

This study utilized RSA and revealed that congruence between elevated parental burnout and permissive parenting styles significantly predicts childhood social anxiety. These findings substantiate the interaction principles of family ecosystem theory, indicating that more attention should be paid to burnout and a permissive household. Consequently, clinical and policy initiatives should implement multidimensional interventions simultaneously strengthening parental psychological resources, regulating family emotional expression, and modifying maladaptive parenting practices to disrupt anxiety transmission pathways.

## Data Availability

The raw data supporting the conclusions of this article will be made available by the authors, without undue reservation.
